# Phenotypic Characterization of the c.1679+1643G>T (1811+1643G>T) Mutation in Hispanic Cystic Fibrosis Patients

**DOI:** 10.3390/children5070091

**Published:** 2018-07-03

**Authors:** Hani K. Fanous, Silvia Delgado-Villata, Reka Kovacs, Eglal Shalaby-Rana, Iman Sami-Zakahri

**Affiliations:** 1 Division of Pulmonary & Sleep Medicine, Children’s National Health System, Washington, DC 20010, USA; princikovacs@gmail.com (R.K.); isami@childrensnational.org (I.S.-Z.); 2 Pediatric Pulmonary & Allergy Division, University of Florida, Gainesville, FL 32608, USA; silviadelgado@ufl.edu; 3 Diagnostic Imaging and Radiology, Children’s National Health System, Washington, DC 20010, USA; ERANA@childrensnational.org

**Keywords:** 1811+1643G>T, c.1679+1643G>T, cystic fibrosis, El-Salvador, Hispanic, phenotype

## Abstract

Cystic fibrosis (CF) is the most common fatal genetic diseases in the United States in Caucasians. More than 2000 genetic mutations have been described and CF is now known to affect other races. The incidence of CF in individuals of Hispanic descent is estimated to be 1:9200. An uncommon mutation, 1811+1643G>T, was recently reported. We report four patients with the 1811+1643G>T mutation (homozygous or heterozygous) and describe their clinical features and compare them to the remainder of our Hispanic cohort group. The homozygous patients had a more severe phenotype compared to the Hispanic cohort in the following areas: their pancreatic status, forced expiratory volume in 1 s (FEV1) and forced vital capacity (FVC), chronic *Pseudomonas aeruginosa* (PA) colonization, pulmonary exacerbations requiring oral and intravenous antibiotics, and hospitalization rate. These preliminary findings suggest that future studies investigating the clinical trajectory with a larger cohort of patients homozygous for the 1811+1643G>T mutation are needed.

## 1. Introduction

Cystic Fibrosis (CF) is one of the most common recessively inherited disorders and was initially described in Caucasian populations. Following the discovery of the CF gene in 1989 and the description of more than 2000 mutations, CF is now known to exist in many racial groups albeit with a lesser frequency. In centers with diverse racial groups the CF Foundation Registry Data reports an increase in minorities from 5 to 8.2% for Latinos, from 3 to 4.6% for African American individuals and from 1.4 to 3.1% for “other” [1].

Cystic fibrosis is caused by a mutation in the gene that regulates the production of the cystic fibrosis transmembrane regulator (CFTR). A lack of or dysfunctional CFTR, affects anion transport in epithelial cells. In the airways this dysfunction alters mucociliary clearance leading to recurrent infections, small airway obstruction and eventual bronchiectasis. Progressive pulmonary damage is the major cause of morbidity and mortality in CF. Other organs that are affected are the pancreas (causing malabsorption and in some subjects cystic fibrosis related diabetes), liver (causing biliary cirrhosis), sweat glands (causing high sweat sodium and chloride levels which cause salt loss, but are also used in diagnosis), and the reproductive tract (causing male infertility) [2].

Traditionally, six classes of mutations have been described, with classes I to III being the most severe, and IV to VI causing less severe disease. In Class I, little to no functional CFTR is produced. Class II mutations are the most common, and cause misfolding of the CFTR protein; the classical example is δ F508. Class III mutations exhibit defective regulation of chloride transport across the apical surface of epithelial cells [3].

The incidence of CF in individual from Hispanic descent has been estimated to be 1:9200 [4]. Numerous mutations have been described, each accounting for a very small percentage, leading to significant genetic heterogeneity. This has made it difficult to not only correlate the clinical phenotype with the gene mutation, but also to classify the mutations. Less than half of Hispanic CF patients carry at least one copy of the δ F508 mutation.

In an effort to shed light on an uncommon CF mutation with a previously undescribed clinical phenotype, we describe the clinical characteristics of an uncommon mutation in four Hispanic patients. The mutation 1811+1.6kbA>G, located in intron 11 of the CFTR gene was first described more than 20 years ago. It is a splicing mutation that occurred mostly in patients of Hispanic descent in the United States, and in patients from South West France in Europe [5]. Individuals who carry the 1811+1.6kbA>G mutation have been reported to have severe CF manifestations as well as pancreatic insufficiency [6]. 

The CFTR sequence variant c.1679+1643G>T, (legacy name 1811+1643G>T) is a recently detected novel mutation. It is also a splicing mutation occurring nine base pairs downstream from the 1811+1.6kbA>G mutation previously described [5]. The 1811+1643G>T mutation was reported in 11 patients who were all of Hispanic descent and frequency of the allele has been estimated to be approximately 1% [7]. The CF center at Children’s National Health System currently follows four patients who carry the 1811+1643G>T mutation. All patients are of Hispanic descent and coincidently are from El-Salvador.

The newly detected mutation, which has only been reported in Hispanic patients, is a novel sequence variant of the 1811+1.6kbA>G mutation in CFTR intron 11, which is known to cause severe CF with pancreatic insufficiency. This is the first description of the clinical phenotype and the level of severity of this mutation. 

## 2. Materials and Methods

Institutional review board (IRB) approval was obtained by the IRB at Children’s National Medical Center, number 3427, on 11 October 2017. Patients with the 1811+1643G>T mutation are described from a retrospective chart review. 

The variables recorded were: birth date; gender; age at diagnosis; clinical diagnoses; genotype; sweat chloride; chronic colonization by *Pseudomonas aeruginosa* (PA) defined as more than 50% of cultures in one year [8]; age of first PA infection; cultures with methicillin resistant *Staphylococcus aureus* (MRSA); age of first MRSA; Mycobacteria cultures; *Burkholderia cepacia* complex cultures; resistant organisms on cultures; history of allergic bronchopulmonary aspergillosis (ABPA), IgE, cystic fibrosis related diabetes (CFRD); pancreatic status, pancreatic-enzyme replacement therapy (PERT) and fecal elastase; body mass index (BMI); forced expiratory volume in one second (FEV_1_) expressed as a percentage predicted; forced vital capacity (FVC) as percent predicted; FEV1/FVC ratio, and forced expiratory flow 25–75 (FEF25–75) as percent predicted. History of meconium ileus; distal intestinal obstruction syndrome (DIOS); pancreatitis; gastroesophageal reflux disease (GERD); use of prokinetic medications were also documented. 

The patients were grouped according to the 1811+1643G>T mutation. Group 1 is homozygous and Group 2 is heterozygous. Note that the two patients in Group 1 are siblings. A third group of 30 CF patients also of Hispanic decent were included. This group was added as a point of reference and not as a control group per se.

Chronic lung pathology attributed to disease progression was assessed on PA and lateral chest X-rays. The most recent X-rays were selected when patients were not having an exacerbation. A blinded pediatric radiologist to the mutation assessed 34 sets using the Brasfield scoring system [9,10,11]. 

Statistical analyses were performed using version 13.0 of the Statistical Package for the Social Sciences (SPSS, IBM, Armonk, NY, USA).

## 3. Results

Group 1 (1811+1643G>T homozygote) included two patients, Group 2 (1811+1643G>T heterozygous) two patients, and Group 3 included 30 patients. See Table 1 and Table 2 for genetic mutations of Group 3. Approximately (7/30) 23.3% of subjects from Hispanic descent are from El-Salvador.

There was no significant association between genotype and gender nor were there any age differences across groups. As displayed in Table 3, patients in Group 1 and 2 had pancreatic insufficiency in comparison to 80% of Group 3. There was also no significant difference across groups in BMI. 

Pulmonary function tests (PFT’s), FEV1, FEV1/FVC, FEF25-75 and FVC were recorded. Results are displayed in Table 4. There was a statistically significant difference between Group 1 and Group 3 for FEV1 (*t* (23) = −2.56, *p* = 0.018) showing that the subjects in Group 1 had significantly lower FEV1 values. Subjects in Group 1 also had a statistically significant lower FVC value compared to both Group 2 and Group 3 (*t* (23) = −2.86, *p* = 0.009).

The Brasfield score can positively correlate with the Shwachman-Kulczycki score and reflect the clinical status of the patient [12].

There was great variability between the homozygous siblings in their total scores (17 and 7) while the heterozygous group has identical scores (15 and 15). Group 3 also had a comparable average score of 18.3, as in Table 5.

## 4. Discussion

A recent study reported that Hispanic patients have worse health outcomes even after adjusting for socio-economic factors [13]. The authors postulated that the causes were likely multifactorial, but one of the potential causes was having an “unclassified” mutation with no widely available treatment options. Hence the importance of this descriptive study characterizing a rare mutation that is “unclassified” in our patients.

The mean age of diagnosis in Group 1 was later (40 months) compared to the other two groups. This may be due to the older sibling’s diagnosis being missed until hospitalization with pneumonia requiring an extensive workup until the CF diagnosis was made, whereas the younger sibling was diagnosed by newborn screening.

Nutritional status was assessed by average BMI or weight for height at enrollment into the study; it was noted that all three groups had similar values. Also, pancreatic insufficiency occurred in 100% of Group 1 and 2 in comparison to only 80% of the reference group. Weight for height was selected as it tends to correlate with lung function.

When PFTs were compared, FEV1 and FVC values in Group 1 were lower than the reference group and statistically significant (in both *p* < 0.05), FEV1/FVC ratio was not different, and FEF 25–75 showed a clinically significant difference although it did not achieve statistical significance.

A total of 41.4% of the reference group cultured PA at least once, Group 1 patients cultured more than once, and Group 2 had no positive PA cultures.

Mean sweat chloride concentrations at diagnosis for Group 1 were compared to the reference group and there was no difference (87.00 and 89.48 mmol/L), while Group 2 had higher values (100.50 mmol/L). This may be due to the fact that in Group 2 the second mutation was a class I and a class II mutation (3876delA and δ F508). There is marked heterogeneity in the expression of mutations classified as class I. In class I, there is a failure of protein synthesis or improper protein folding/trafficking to the cell membrane respectively resulting in no or little CFTR at the cell surface and consequently no or minimal chloride transport. It is unknown whether this mutation in particular has any influence on modifier genes that influence the severity of the clinical manifestations.

Group 1 had more exacerbations (3.3/year) than both Group 2 and reference groups (1/year and 1.3/year, respectively). They also had more admissions/year (1.4/year compared with 0.6/year and 0.6/year for Group 2 and reference groups).

Chest X-rays were assessed using the Brasfield scoring system. A lower score indicates more severe lung disease. Interestingly the two patients who were homozygous had two significantly different scores even though they are siblings. One had a very low score (worse score) and the other had a higher score (better score) which was comparable to the mean score of reference group. The variability in severity of diseases between the two siblings is perplexing. It is worth noting that the younger sibling with the more “severe” progression of the disease was diagnosed at a younger age than the older sibling.

Our study has limitations; primarily, the small sample size of the patients with the homozygous and heterozygous gene mutation, as the prevalence of this mutation in the CF population is quite rare. The small sample size prevented us from performing parametric testing comparing Groups 1 and 2 to the reference group as a control group. Other limitations include the fact that the two patients who were homozygous are also siblings, therefore, it is difficult to separate the effect of non-genetic influences on CF outcomes.

In summary, the newly detected mutation 1811+1643G>T is a novel sequence variant of the 1811+1.6kbA>G mutation in CFTR intron 11. Patients who are homozygous for 1811+1643G>T mutation displayed a severe phenotype as evidenced by their pancreatic status, poor pulmonary function, chronic colonization with PA, large number of pulmonary exacerbations requiring the use of oral and intravenous antibiotics, as well as hospitalizations. They did not appear to be different from other Hispanic subjects with CF in terms of their nutritional status, radiological findings on chest X-ray, or sweat chloride values.

Future studies with a larger cohort of patients homozygous for the 1811+1643G>T mutation are needed, to determine their clinical trajectory, and level of severity compared with other Hispanic patients with CF with different mutations.

## Figures and Tables

**Table 1:** Phenotypic variables for all CF mutations by group.

	Group 1	Group 2	Group 3
	1811+1643G>T Homozygous	1811+1643G>T Heterozygous	Hispanic CF Reference Group
Patients (*n*)	2	2	30
Sex (male/female)	0/2	1/1	21/9
Average age in months	132.09	64.41	122.83
Average age at diagnosis in months (SD)	39.89 (54.55)	2.58 (1.42)	18.44 (43.73)
Positive cultures for PA (yes/no)	1/1	0/2	12/17
Average BMI (kg/m^2^) (SD)	17.29 (1.56)	15.75 (1.34)	18.48 (3.82)
Positive cultures of *Burkholderia cepacia* (yes/no)	0/2	0/2	0/30
Sweat chloride concentration (mmol/L) (SD)	87.00 (2.83)	100.50 (3.54)	89.48 (22.46)
Pancreatic insufficiency (yes/no)	2/0	2/0	24/6

CF, cystic fibrosis; SD, standard deviation; PA, *Pseudomonas aeruginosa*; BMI, body mass index.

**Table 2:** Breakdown of Group 3 CF Mutations.

Mutation1/Mutation2	Number of Subjects
N1303K/Unknown	1
F508del/3876delA	7
3120+1G->A/R1066C	1
S549N/TG12/T5	1
F508del/1949del84	1
F508del/Unknown	3
F508del/F508del	6
Unknown/Unknown	3
F508del/P205S	1
3876delA/3876delA	2
G542X/360-365insT	1
F508del/R553X	1
S549N/S549N	1
TG11/NT3878	1

**Table 3:** Characteristics of the four CF patients and the Hispanic CF Reference Group.

	1811+1643G>T Homozygous *n* = 2		1811+1643G>T Heterozygous *n* = 2		Hispanic CF Group *n* = 30 M (SD)
	Patient 1	Patient 2	Patient 1	Patient 2	
Sex	Female	Female	Male	Female	
Age at diagnosis (months)	78.5	1.3	1.5	3.6	6.7 (9.0)
Sweat test (mmol/L)	89.0	85.0	98.0	103.0	88.2 (22.5)
Average number of exacerbations/year	2.3	4.3	0.5	1.5	1.2 (0.9)
Average number of admissions/year	0.50	2.30	0.75	0.50	0.50 (0.40)
Age at first exacerbation (months)	104.20	1.80	15.97	16.56	26.70 (2.60)
Age at first admission (months)	106.9	5.1	2.5	35	26.7 (3.0)
Hemoptysis	No	Yes	No	No	5 (16.7%) Yes
Clubbing	Yes	Yes	Yes	Yes	21 (70%) Yes
DIOS	No	Yes	No	No	5 (16.7%) Yes
PI	Yes (PE1 < 100)	Yes (PE1 < 100)	Yes (PE1 < 100)	Yes (PE1 < 100)	24 (80%) Yes
Gastrostomy	No	Yes	No	No	2 (6.7%) Yes

M, mean; DIOS, distal intestinal obstruction syndrome; PI, pancreatic insufficiency; PE, pancreatic fecal elastase.

**Table 4:** Pulmonary status for all groups (% predicated value).

		Group 1	Group 2	Group 3
		1811+1643G>T Homozygous	1811+1643G>T Heterozygous	Hispanic CF Reference Group
FEV_1_ *	Mean	46.50 *	73.00	84.91 *
	SD	16.263	9.899	20.805
**FEV_1_/FVC**	Mean	81.50	83.50	79.59
	SD	3.536	23.335	9.630
**FEF 25**–**75**	Mean	37.50	77.50	67.32
	SD	20.506	58.690	32.079
FVC *	Mean	57.50 *	81.00	95.36 *
	SD	21.920	9.899	17.513

* Significant at *p* < 0.05 level; FEV_1_, forced expiratory volume in 1 s; FVC, forced vital capacity; FEF 25–27, forced expiratory flow 25–75.

**Table 5:** Brasfield chest radiograph score by group.

	1811+1643G>T Homozygous *n* = 2		1811+1643G>T Heterozygous *n* = 2		Hispanic CF Group *n* = 30 Rating (Majority %)
	Patient 1	Patient 2	Patient 3	Patient 4	
Air trapping	2	3	3	1	0 (62%)
Linear markings	2	4	3	2	2 (34%)
Nodular cystic lesions	2	4	2	2	0 (48%)
Large lesions	0	3	0	2	0 (65%)
General severity	2	4	2	2	2 (34%)
Final score total	17	7	15	15	18.3 (5.5%) *

* Group 3 M (SD).
